# Associations between socio-demographics, nutrition knowledge, nutrition competencies and attitudes in community-dwelling healthy older adults in Singapore: findings from the SHIELD study

**DOI:** 10.1186/s41043-021-00277-4

**Published:** 2021-12-11

**Authors:** Rebecca Hui San Ong, Wai Leng Chow, Magdalin Cheong, Gladys Huiyun Lim, Weiyi Xie, Geraldine Baggs, Dieu Thi Thu Huynh, Hong Choon Oh, Choon How How, Ngiap-Chuan Tan, Siew Ling Tey, Samuel Teong Huang Chew

**Affiliations:** 1 Health Services Research, Changi General Hospital, Singapore, Singapore; 2 Department of Dietetic and Food Services, Changi General Hospital, Singapore, Singapore; 3 Abbott Nutrition Research and Development, Columbus, OH USA; 4 Abbott Nutrition Research and Development, Asia-Pacific Center, Singapore, Singapore; 5 Care and Health Integration, Changi General Hospital, Singapore, Singapore; 6 SingHealth Polyclinics, Singapore, Singapore; 7 Department of Geriatric Medicine, Changi General Hospital, Singapore, Singapore

**Keywords:** Nutrition knowledge, Nourished, Community-dwelling, Older adults, Cross-sectional study

## Abstract

**Background:**

Nutrition literacy refers to an individual’s knowledge, motivation and competencies to access, process and understand nutrition information to make nutrition-related decisions. It is known to influence dietary habits of individuals including older adults. This cross-sectional study was designed to: (1) understand the nutrition knowledge, competencies and attitudes of community-dwelling older adults in Singapore, (2) examine the differences between their nutrition knowledge, and socio-demographic factors, competencies and attitudes and (3) identify factors associated with better nutrition knowledge in older healthy adults in Singapore.

**Methods:**

A total of 400 (183 males and 217 females) nourished community-dwelling older adults aged 65 years and above took part in this study. Malnutrition Universal Screening Tool (MUST) was used to determine individuals who were at low risk of undernutrition. Nutrition knowledge, competencies, attitudes and sources of nutrition information were measured using a locally developed scale. Nutrition knowledge scores were summed to form the nutrition knowledge index (NKI). Associations between NKI, competencies, attitudes and socio-demographic variables were examined using Chi-square and Fisher’s exact tests. Factors associated with NKI were determined using a stepwise regression model with resampling-based methods for model averaging.

**Results:**

Bivariate analyses found significant differences in NKI scores for gender, monthly household earnings, type of housing, the self-reported ability to seek and understand nutrition information and having access to help from family/friends. Females had higher NKI scores compared to males (*p* < 0.001). Compared to females, more males left food decisions to others (*p* < 0.001), and fewer males reported consuming home-cooked food (*p* = 0.016). Differences in educational level were found for competencies like the self-reported ability to seek (*p* < 0.001) and verify nutrition information (*p* < 0.001). Stepwise regression analysis showed that being female, Chinese, self-reported ability to understand nutrition information and having access to help from family/friends were associated with higher NKI scores.

**Conclusions:**

Our study revealed that nutrition knowledge of older males in Singapore was lower than females and more left food decisions to others. Nutrition education programs could be targeted at both the older male, their caregivers and minority ethnic groups.

*Trial Registration* This study was registered on 7 August 2017 at clinicaltrials.gov (ref. NCT03240952).

## Background

Singapore is a multi-ethnic urban city state with the second-fastest aging population in Asia [1]. The proportion of older adults in Singapore aged 65 years and above has increased from 8.8% in 2008 to 14.4% in 2019 [2]. In Singapore, about 33% of community-dwelling older adults aged 65 to 74 years old were at risk of poor nutritional status [3]. This proportion increased to about 41.0% for older adults aged 75 years and above [3]. For older adults, it is well recognized that compromised nutrition is associated with adverse clinical and functional outcomes like physical frailty, higher mortality, longer inpatient stays and increased intensive care need [4–7].

Nutrition literacy is considered a specific domain of health literacy by several researchers [8–12] and its definition is derived from existing health literacy definitions [13]. Several authors have defined nutrition literacy as the extent to which individuals are able to attain, assess and understand basic nutrition information required to make appropriate nutrition-related decisions [8, 9, 11, 12, 14, 15]. Key elements of nutrition literacy include nutrition knowledge, competencies and attitudes [16].

Although associations between nutrition literacy and health related outcomes have not been established in existing literature, studies have shown that elements of nutrition literacy can influence dietary habits. Wardle and colleagues reported that nutrition knowledge plays a pivotal role in healthy dietary habits and partially mediates socio-demographic variations in dietary intakes [17]. A meta-analysis reported a significant, albeit weak positive association between nutrition knowledge and healthy eating [18]. It is also well recognized that healthy eating in older adults is multi-faceted and shaped by both individual and collective factors [19–23]. An individual’s competency in obtaining, understanding nutrition information and knowing how to access nutrition health services can have an impact on the nutrition knowledge of the individual. Studies have reported that the lack of aforementioned skills may contribute to poor nutrition health [16, 24]. Nutrition attitudes are centered on the perceptions and beliefs influencing food choices [19, 20], mediating the link between nutrition knowledge and dietary habits [25]. A systematic review reported associations between better nutrition knowledge, positive dietary attitudes and healthy dietary habits [26]. This is supported by more recent research noting similar associations within Asian and Western older adults [20, 27].

Socio-demographic factors such as higher educational qualification were associated with better nutrition knowledge [28] and positive nutrition attitudes in older adults [20, 29]. Gender differences were also reported to be associated with nutrition knowledge, with some studies reporting older adult females to have higher nutrition knowledge scores than males [30, 31], and other studies noting the opposite [20, 28].

There are several published instruments in existing literature which measure levels of nutrition knowledge, competencies and attitudes of participants. However, these instruments vary widely and were mostly either developed for Caucasians or validated in a predominantly Caucasian population [14]. This is important as there is a cultural aspect to nutrition literacy and behavior [16, 32, 33]. Given the cultural diversity of the Asian population, it is important to develop instruments that are tailored to each culture and group. Aihara and Minai previously developed a nutrition literacy instrument for older Japanese people [30]; however, items were based on dietary guidelines specific to Japan. The nutrition knowledge, attitudes and behavior scale in Taiwan’s Elderly Nutrition and Health Survey consisted of questions on Chinese traditional beliefs on food (“heaty” or “cooling”) and food-texture [20], which might not be applicable to older adults of different ethnicities. There is no instrument that is currently relevant for Singapore’s multi-ethnic population; hence, the Nutrition Literacy Questionnaire (NLQ) was developed for the purpose of this study.

To encourage healthier eating habits and to design nutrition educational interventions for older adults, it is crucial to understand their current levels of nutrition knowledge, attitudes and competencies gaps and to understand factors associated with nutrition knowledge. However, there is a dearth of local studies that examine the nutrition knowledge of older adults. Hence, the overall aim of this cross-sectional study is to understand the nutrition knowledge, competencies and attitudes of community-dwelling older adults aged 65 years and above in Singapore. The secondary aims are to: (1) examine the associations between nutrition knowledge, and socio-demographic factors, competencies (e.g., ability to obtain and understand information on nutrition) and attitudes and (2) identify factors associated with better nutrition knowledge.

## Methods

### Study design and participants

This cross-sectional study was part of the Strengthening Health In ELDerly through nutrition (SHIELD) study.. Convenience sampling was used to recruit participants between September 2017 and January 2018 from four study sites located in the eastern part of Singapore, namely Changi General Hospital (CGH), Bedok Polyclinic, Marine Parade Polyclinic and Tampines Polyclinic. Participants were eligible for this study if they met the following criteria: (i) aged 65 years and above, (ii) Malnutrition Universal Screening Tool (MUST) score was 0, (iii) a community dweller (i.e., not discharged to or residing in a residential intermediate and long-term care facility), (iv) community ambulant with or without aid and (v) able to communicate and follow instructions. Participants with any stable chronic disease(s), defined as a controlled disease state [34], and which were not listed under the exclusion criteria were also eligible for the study. Participants were ineligible for the study if they were diagnosed with any of the following: (i) type 1 or type 2 diabetes mellitus, (ii) dementia, (iii) active infectious disease (tuberculosis, hepatitis B or C, HIV infection), (iv) severe gastrointestinal disorders (celiac disease, short bowel syndrome, pancreatic insufficiency or cystic fibrosis), (v) end-stage organ diseases or pre-terminal diseases, (vi) acute myocardial infarction within the last 30 days or (vii) any active malignancy within the last five years. This study was approved by SingHealth Centralised Institutional Review Board (ref no. 2017/2273) and was registered at clinicaltrials.gov (ref. NCT03240952).

### Procedures and measures

Following enrollment, all study participants were requested to attend one scheduled visit at Changi General Hospital on a separate day. Nutritional status of participants was assessed by trained study personnel using the Malnutrition Universal Screening Tool (MUST) [35]. The MUST consists of three main scoring components, namely body mass index (BMI), recent unplanned weight loss and effects of acute disease. Low risk of undernutrition was defined as MUST score = 0 [36, 37]. BMI was calculated to the nearest 0.1 kg/m^2^. Height was measured to the nearest 0.01 m, and weight was measured to the nearest 0.1 kg using a weight and height machine (Avamech B1000) calibrated according to the manufacturer’s specifications. Information on medical history, socio-demographics (i.e., age, sex, ethnicity, education, marital status, living arrangements, residential type and financial situation) and lifestyle (i.e., smoking, alcohol consumption) of the participants was also collected via self-administered questionnaires during the study visit. Onsite study personnel checked the questionnaires for completeness and if necessary, clarified any missing data with participants on the spot. Age was categorized into < 75 or ≥ 75 years. Education was categorized into primary, secondary, pre-university and university and above. Information on physical health was obtained through the Charlson Comorbidity Index (CCI) [38] and Modified Barthel Index (MBI) instruments [39].

### Nutrition knowledge, competencies and attitudes

The NLQ was developed using evidence from published literature, nutritional knowledge materials published by the local health promotion agency [40] and expert inputs. The instrument was piloted and tested among five older adults and two dietitians to determine face validity. It was found to be acceptable. The instrument was then translated and back-translated into Chinese and Malay, the two other most commonly used languages in Singapore.

The NLQ consisted of three main components namely nutrition knowledge, competencies and attitudes. Sources of nutrition information and factors influencing food choices were also collected and analyzed separately. Firstly, nutrition knowledge of participants was assessed based on their knowledge of local nutritional recommendations outlined in The Recipe For Healthy Ageing: Nutrition Guide [40]. There were 7 multiple choice questions, each question had three possible answers to choose from, and there was only one correct answer. Questions covered food groups and recommended portion sizes. A score of 1 was assigned for a correct response and 0 for otherwise. Scores were summed to form the nutrition knowledge index (NKI). Total scores ranged from 0 to 7, with higher scores indicating better knowledge level. Secondly, nutrition competencies of participants were assessed using 9 binary questions. For example, “Do you know where to go to find information about nutrition? (Yes/No)”. Thirdly, nutrition attitudes of participants were measured using 4 statements (e.g., “It is not important to eat nutritious meals at my age”) related to their dietary habits and the participants were asked to rate each of these statements on a four-point Likert scale (4 = Strongly Agree, 3 = Agree, 2 = Disagree, 1 = Strongly Disagree). Participants could choose more than one option(s) for sources of nutrition information and factors influencing their food choices.

### Sample size and statistical analysis

All participants who had a MUST score of 0 (*N* = 400) from the larger SHIELD study were included for this cross-sectional study. All analyses were performed using SAS version 9.4 (SAS Institute, Cary, NC, USA), and significance level was set at 0.05. The responses on the Likert scale were also re-categorized. “Strongly agree” and “Agree” were categorized together as “Agree”, while “Strongly disagree” and “Disagree” were categorized together as “Disagree”. The option “Refused to answer/don’t know” for monthly household earnings (SGD) was excluded from analyses. Chi-square and, where appropriate, Fisher’s exact tests were used for tests of associations between nutrition knowledge, competencies, attitudes and socio-demographic factors. Stepwise multiple linear regression modeling was used to examine the factors associated with the nutrition knowledge index. Bivariate analyses results were taken into consideration together with literature in this area for variables selection. Model inputs consisted of 20 potential variables. The dependent variable was the nutrition knowledge index, the independent variables consisted of socio-demographic factors, nutrition competencies and nutrition-related attitudes (Table 1). Resampling-based methods for model averaging were used to mitigate for known limitations of stepwise selection including selection bias in parameter estimates and reliance on a single best model [41].

**Table 1:** Independent variables

Main categories	Variables
Socio-demographic factors	Gender Ethnicity Education level Marital status Living arrangements Caregiving arrangements Residences type
Nutrition competencies	*Binary answers (e.g., yes or no)* Aware of where to seek nutrition information Able to understand nutrition information Able to verify nutrition information Leaves food decision to others Consumption of home cooked meals Has access to help from family/friends Able to access health services Comfortable sharing dietary information with healthcare professionals
Nutrition-related attitudes	*Rated on a four-point Likert scale (4* = *Strongly Agree, 3* = *Agree, 2* = *Disagree, 1* = *Strongly Disagree)* Not important to consume nutritious meals Prefer to eat at home Consumption of simpler meals when alone/with spouse Cease consumption of unhealthy favourite foods

## Results

### Participant characteristics

The demographic profile of the study sample is shown in Table 2. Of the 400 participants, 217 (54.2%) were females. Majority were married (74.2%), of Chinese ethnicity (83.0%) and had education levels of secondary level and above (84.5%) with a mean age of 71.2 years. Majority were independent according to MBI (93.0%) and a Charlson Comorbidity score of 0 (98.0%).

**Table 2:** Characteristics of study participants

	*n*	%
*Gender*		
Male	183	45.8
Females	217	54.3
*Age categories*		
< 75 years	301	75.2
≥ 75 years	99	24.8
*Ethnicity*		
Chinese	332	83.0
Others	68	17.0
*Marital status*		
Married	297	74.2
Single	103	25.8
*Highest level of education*		
Primary	62	15.5
Secondary	186	46.5
Pre-university	100	25.0
University and above	52	13.0
*Smoking status*		
Non-smoker	333	83.3
Daily/occasional smoker	10	2.5
Past smoker	57	14.3
*Alcohol consumption in the last 12 months*		
No alcohol	287	71.8
< Once a month	69	17.3
≥ Once a month	44	11.0
*Modified Barthel Index*		
Moderate dependence (60–79)	7	1.8
Slight dependence (80–99)	21	5.3
Independent (100)	372	93.0
*Charlson Comorbidity Score*		
0	392	98.0
1	6	1.5
2	1	0.3
3	1	0.3
*Household earnings/month (SGD)*		
< 2000	166	41.5
2000–3999	66	16.5
4000–5999	32	8.0
6000 and above	38	9.5
Refused to answer/don’t know	98	24.6
*Housing type*		
1–3 room public flats	74	18.5
4–5 room public flats	186	46.5
Private properties and others	140	35.0

### Socio-demographic factors and nutrition knowledge, competencies and attitudes

Gender differences between nutrition knowledge, competencies and attitudes are shown in Table 3. Compared to males, more females correctly identified portion sizes for wholegrains (58.1% vs. 38.3%; *p* < 0.001) and proteins (*p* = 0.02). More females made their own food decisions (*p* < 0.001) and consumed home-cooked food compared to males. More males agreed that they would eat simpler meals if alone/with spouse (55.2% vs. 43.8%; *p* = 0.02). In addition, most participants agreed with the importance of consuming nutritious meals (91.1%) and would cease consumption of their favorite foods if they discovered them to be unhealthy (82.8%). Majority preferred eating home-cooked meals (94.5%), although 196 (49%) agreed that they were more likely to eat simpler meals if alone/with spouse (Table 3).

**Table 3:** Nutrition knowledge, competencies and attitudes by gender

	Total (*n* = 400) *n* (%)	Male (*n* = 183) *n* (%)	Female (*n* = 217) *n* (%)	*p* value between gender
**Nutrition knowledge**				
*Food type—wholegrains, n (%)*				
Incorrect	41 (10.2)	22 (12.0)	19 (8.8)	0.283
Correct	359 (89.8)	161 (88.0)	198 (91.2)	
*Food type—fruits/vegetables, n (%)*				
Incorrect	4 (1.0)	3 (1.6)	1 (0.5)	0.336
Correct	396 (99.0)	180 (98.4)	216 (99.5)	
*Food type—meat, n (%)*				
Incorrect	3 (0.7)	1 (0.5)	2 (0.9)	1.000
Correct	397 (99.3)	182 (99.5)	215 (99.1)	
*Drink type, n (%)*				
Incorrect	5 (1.2)	4 (2.2)	1 (0.5)	0.183
Correct	395 (98.8)	179 (97.8)	216 (99.5)	
*Portion—wholegrains, n (%)*				
Incorrect	204 (51.0)	113 (61.7)	91 (41.9)	< 0.001
Correct	196 (49.0)	70 (38.3)	126 (58.1)	
*Portion – fruits/vegetables, n (%)*				
Incorrect	167 (41.7)	86 (47.0)	81 (37.3)	0.051
Correct	233 (58.3)	97 (53.0)	136 (62.7)	
*Portion—protein, n (%)*				
Incorrect	130 (32.5)	70 (38.3)	60 (27.6)	0.024
Correct	270 (67.5)	113 (61.7)	157 (72.4)	
**Nutrition competencies**				
*Aware of where to seek nutrition information*				
a. Yes	337 (84.3)	149 (81.4)	188 (86.6)	0.154
b. No	63 (15.7)	34 (18.6)	29 (13.4)	
*Able to understand nutrition information*				
a. Yes	376 (94.0)	172 (94.0)	204 (94.0)	0.993
b. No	24 (6.0)	11 (6.0)	13 (6.0)	
*Able to verify nutrition information*				
a. Yes	196 (49.0)	89 (48.6)	107 (49.3)	0.953
b. No	204 (51.0)	94 (51.4)	110 (50.7)	
*Leaves food decision to others*				
a. Yes	101 (25.3)	78 (42.6)	23 (10.6)	< 0.001
b. No	299 (74.7)	105 (57.4)	194 (89.4)	
*Consumption of home cooked meals*				
a. Yes	361 (90.3)	158 (86.3)	203 (93.5)	0.016
b. No	39 (9.7)	25 (13.7)	14 (6.5)	
*Has access to help from family/friends*				
a. Yes	366 (91.5)	166 (90.7)	200 (92.2)	0.603
b. No	34 (8.5)	17 (9.3)	17 (7.8)	
*Able to access health services*				
a. Yes	395 (98.8)	180 (98.4)	215 (99.1)	0.664
b. No	5 (1.2)	3 (1.6)	2 (0.9)	
*Comfortable sharing dietary information with healthcare professionals*				
a. Yes	391 (97.8)	180 (98.4)	211 (97.2)	0.517
b. No	9 (2.2)	3 (1.6)	6 (2.8)	
**Nutrition-related attitudes**				
*Not important to consume nutritious meals*				
a. Agree	36 (9.1)	18 (9.9)	18 (8.3)	0.592
b. Disagree	364 (90.9)	165 (90.1)	199 (91.7)	
*Prefer to eat at home*				
a. Agree	378 (94.5)	169 (92.3)	209 (96.3)	0.083
b. Disagree	22 (5.5)	14 (7.7)	8 (3.70)	
*Consumption of simpler meals when alone/with spouse*				
a. Agree	196 (49.0)	101 (55.2)	95 (43.8)	0.023
b. Disagree	204 (51.1)	82 (44.8)	122 (56.2)	
*Cease consumption of unhealthy favourite foods*				
a. Agree	331 (82.8)	149 (81.4)	182 (83.9)	0.518
b. Disagree	69 (17.3)	34 (18.6)	35 (16.1)	

Table 4 shows nutrition knowledge index by socio-demographic factors, competencies and attitudes. A higher nutrition index was noted for females, those who reported and average household earnings of less than SGD$2000, individuals who lived in private properties. For nutrition-related competencies, a higher NKI score was noted for individuals who self-reported an ability to seek nutrition information (*p* = 0.011), understood nutrition information (*p* = 0.008), did not leave food decisions to others (*p* = 0.012), had access to help from family or friends (*p* = 0.009) and did not consume simpler meals when alone/or only with spouse (*p* = 0.016).

**Table 4:** Associations between nutrition knowledge, socio-demographics, competencies and attitudes

	*n* (%)	Nutrition knowledge index (mean, SE)	*p* value
*Gender*			
a. Male	183 (45.8)	5.37 (0.07)	< 0.001
b. Female	217 (54.3)	5.82 (0.07)	
*Marital status*			
a. Married	297 (74.3)	5.56 (0.06)	0.079
b. Single	103 (25.7)	5.77 (0.10)	
*Household earnings/month (SGD)*			
a. < 2000	166 (41.5)	5.76 (0.08)	
b. 2000–3999	66 (16.5)	5.33 (0.12)	0.035
c. 4000–5999	32 (8.0)	5.66 (0.18)	
d. 6000 and above	38 (9.5)	5.55 (0.16)	
*Housing type*			
a. HDB 1–3 room flats	74 (18.5)	5.73 (0.12)	
b. HDB 4–5 room flats	186 (46.5)	5.47 (0.07)	0.026
c. Private properties	140 (35.0)	5.75 (0.09)	
*Education level*			
a. Primary	62 (15.5)	5.66 (0.13)	
b. Secondary	186 (46.5)	5.54 (0.07)	0.406
c. Pre-university	100 (25.0)	5.62 (0.10)	
d. University and above	52 (13.0)	5.81 (0.14)	
*Living arrangements*			
a. Alone	35 (8.8)	5.54 (0.17)	
b. With immediate family	336 (84.0)	5.61 (0.06)	0.776
c. With helpers/friends/extended family	29 (7.2)	5.72 (0.19)	
*Caregiving arrangements*			
a. Alone	54 (13.5)	5.56 (0.14)	
b. With immediate family	208 (52.0)	5.52 (0.07)	0.072
c. With helpers/friends/extended family	138 (34.5)	5.78 (0.19)	
*Aware of where to seek nutrition information*			
a. Yes	337 (84.3)	5.67 (0.06)	0.011
b. No	63 (15.7)	5.32 (0.13)	
*Able to understand nutrition information*			
a. Yes	376 (94.0)	5.65 (0.05)	0.008
b. No	24 (6.0)	5.08 (0.21)	
*Able to verify nutrition information*			
a. Yes	196 (49.0)	5.64 (0.07)	0.764
b. No	204 (51.0)	5.63 (0.11)	
*Leaves food decision to others*			
a. Yes	101 (25.3)	5.40 (0.10)	0.012
b. No	299 (74.7)	5.69 (0.06)	
*Consumption of home cooked meals*			
a. Yes	361 (90.3)	5.62 (0.05)	0.622
b. No	39 (9.7)	5.54 (0.16)	
*Has access to help from family/friends*			
a. Yes	366 (91.5)	5.66 (0.05)	0.009
b. No	34 (8.5)	5.18 (0.17)	
*Able to access health services*			
a. Yes	395 (98.8)	5.62 (0.05)	0.175
b. No	5 (1.2)	5.00 (0.46)	
*Comfortable sharing dietary information with healthcare professionals*			
a. Yes	391 (97.8)	5.62 (0.05)	0.612
b. No	9 (2.2)	5.44 (0.34)	
*Not important to consume nutritious meals*			
a. Agree	36 (9.1)	5.31 (0.17)	0.056
b. Disagree	364 (90.9)	5.65 (0.05)	
*Prefer to eat at home*			
a. Agree	378 (94.5)	5.63 (0.05)	0.160
b. Disagree	22 (5.5)	5.32 (0.22)	
*Consumption of simpler meals when alone/with spouse*			
a. Agree	196 (49.0)	5.49 (0.07)	0.016
b. Disagree	204 (51.1)	5.74 (0.07)	
*Cease consumption of unhealthy favourite foods*			
a. Agree	331 (82.8)	5.62 (0.06)	0.955
b. Disagree	69 (17.3)	5.61 (0.12)	

Table 5 shows the associations of socio-demographic factors with competencies and attitudes. Compared to those with primary level education or below, more individuals with higher educational qualification reported having the ability to seek (*p* < 0.001) and verify nutrition information (*p* < 0.001). Compared to currently married individuals, those who were single were less likely to report leaving food decisions to others (*p* = 0.002). Compared to those who lived alone, those who were living with others were more likely to report leaving food decisions to others (*p* = 0.004). In terms of caregiving arrangements, for older adults who reported self-caregiving, majority (81.0%) made their own food decisions and was able to access healthcare services (94.0%). However, they reported lower rates of consuming home-cooked food compared to those who were cared for by family or others (*p* = 0.011).

**Table 5:** Significant associations between socio-demographic factors, nutrition competencies and attitudes

	Education (%)					Marital status (%)			Living arrangements (%)				Caregiving arrangements (%)			
	Pri (*n* = 62)	Sec (*n* = 186)	Pre-Uni (*n* = 100)	Uni and above (*n* = 52)	*p* value	Single (*n* = 103)	Married (*n* = 297)	*p* value	Alone (*n* = 35)	Family (*n* = 336)	Helpers/friends/extended family (*n* = 29)	*p* value	Self (*n* = 54)	Family (*n* = 208)	Helpers/friend/extended Family (*n* = 138)	*p* value
*Aware of where to seek nutrition information*																
a. Yes	63.0	85.0	88.0	98.0	< 0.001	–	–	–	–	–	–	–	–	–	–	–
b. No	37.0	15.0	12.0	2.0												
*Able to understand nutrition information*																
a. Yes	89.0	93.0	96.0	100	0.049	–	–	–	–	–	–	–	–	–	–	–
b. No	11.0	7.0	4.0	0												
*Able to verify nutrition information*																
a. Yes	10.0	49.0	63.0	67.0	< 0.001	–	–	–	–	–	–	–	–	–	–	–
b. No	54.8	18.3	15.0	11.5												
c. Trust others	35.5	32.3	22.0	21.2												
*Leaves food decision to others*																
a. Yes	–	–	–	–	–	14.0	29.0	0.002	3.0	28.0	21.0	0.004	19.0	33.0	17.0	0.002
b. No						86.0	71.0		97.0	72.0	79.0		81.0	67.0	83.0	
*Consumption of home cooked meals*																
a. Yes	–	–	–	–	–	–	–	–	–	–	–	–	80.0	93.0	90.0	0.011
b. No													20.0	7.0	10.0	
*Has access to help from family/friends*																
a. Yes	85.0	90.0	97.0	94.0	0.047	–	–	–	–	–	–	–	–	–	–	–
b. No	15.0	10.0	3.0	6.0												
*Able to access health services*																
a. Yes	–	–	–	–	–	–	–	–	94.0	99.0	97.0	0.031	94.0	100.0	99.0	0.031
b. No									6.0	1.0	3.0		6.0	0.0	1.0	
*Cease consumption of unhealthy favourite foods*																
a. Agree	87.0	87.0	81.0	67.0	0.009	–	–	–	–	–	–	–	–	–	–	–
b. Disagree	13.0	13.0	19.0	33.0												

Pri: primary, sec: secondary, uni: university

**Fig. 1:**
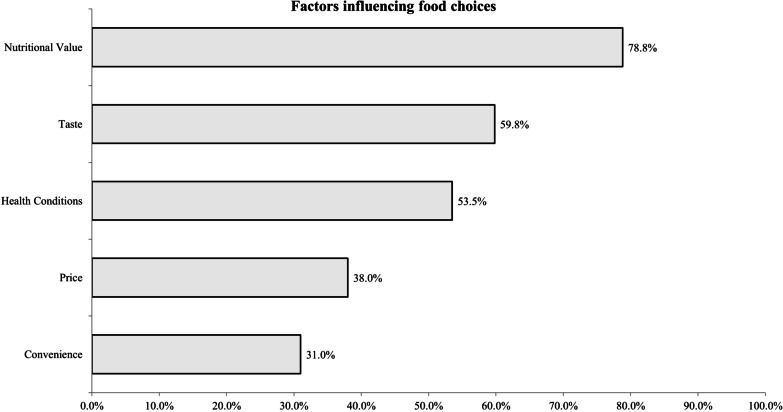
Self-reported factors influencing individual's food choices

Most participants reported nutritional value influenced their food choices (78.8%), and this was followed by taste (59.8%) and health conditions (53.5%) (Fig. 1).

The results of the stepwise regression are shown in Table 6; it was observed that being female (*p* < 0.001), of Chinese ethnicity (*p* = 0.026), self-reported ability to understand nutrition information (*p* = 0.035) and having access to help from family/friends (*p* = 0.051) was associated with higher NKI.

**Table 6:** Factors associated with nutrition knowledge index

Model	Unstandardized coefficients		
	β	Std. error	*p* value
*Gender*			
Males	0		
Females	0.4213	0.09924	< 0.001
*Ethnicity*			
Non-Chinese	0		
Chinese	0.4213	0.09924	0.026
*Able to understand nutrition information*			
Yes	0		
No	− 0.4469	0.2115	0.035
*Has access to help from family/friends*			
Yes	0		
No	− 0.3531	0.1802	0.051

### Sources of nutritional information

**Fig. 2:**
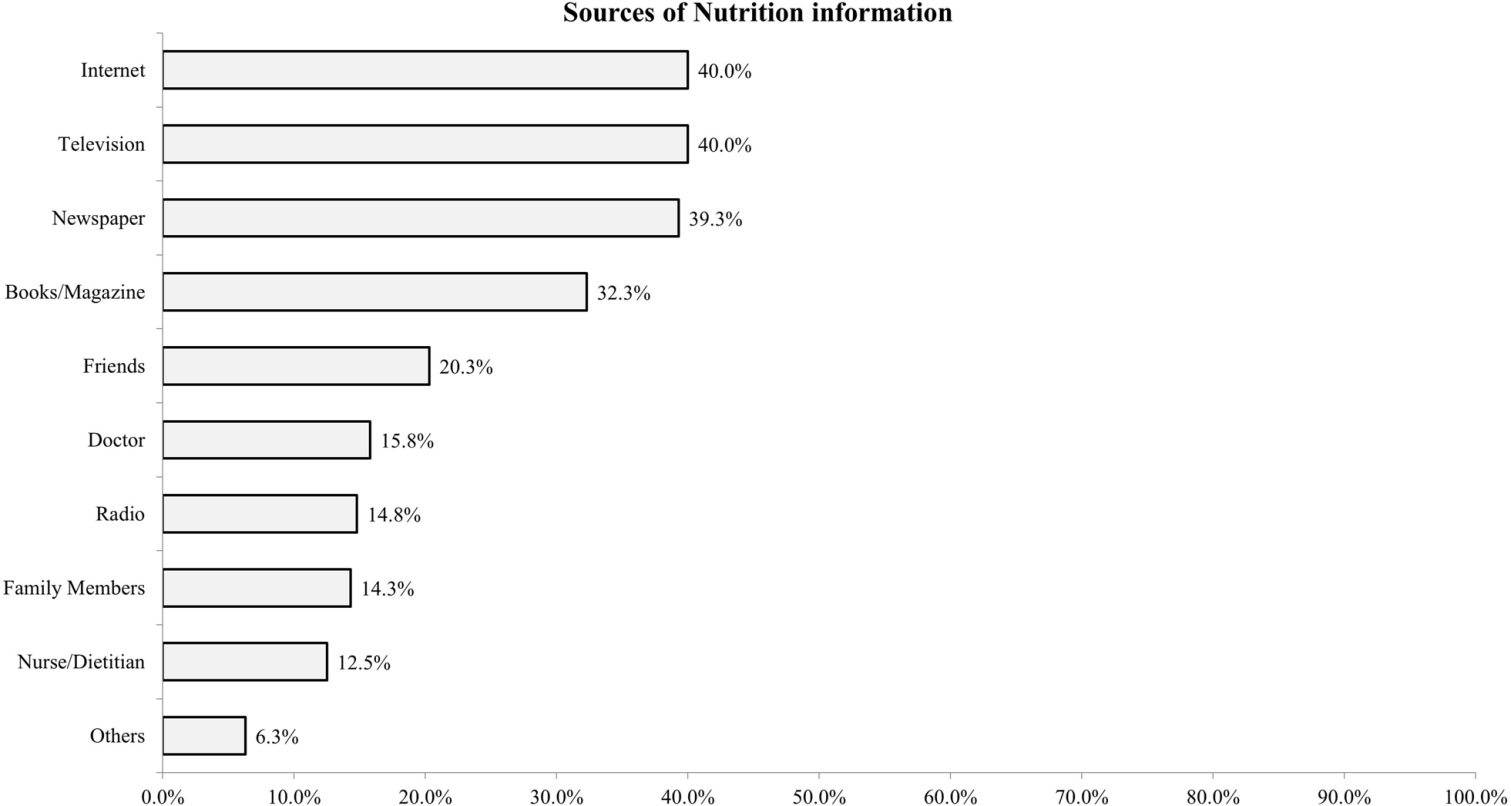
Sources of Nutrition information

Figure 2 shows the most commonly cited sources of nutrition information. Among the study participants, the most commonly cited source(s) was the television (40.0%), internet (40.0%), followed by traditional print media such as newspapers (39.3%), books/magazines (32.3%) and word of mouth like through friends (20.3%) and family (14.3%). Compared to males, females were significantly more likely to turn to television (45.2% vs. 33.9%), radio (19.8% vs. 8.7%) and friends (24.4% vs. 15.3%) for nutrition information.

## Discussion

To the best of our knowledge, this is the first study of its kind to examine the nutrition knowledge, competencies and attitudes among relatively healthy older adults, and the results might be useful for guiding nutrition strategies in a multi-ethnic population. Gender, ethnicity, the self-reported ability to understand nutrition information and having access to help from family and friends are significant factors associated with nutrition knowledge of the older adult population with normal nutrition status.

Though some studies have been conducted on nutrition literacy among Asian older adults [20, 30], their instruments were specially designed for their respective local populations. Thus, it is difficult to compare the nutrition literacy levels of our participants with that of other studies. We found nutrition knowledge and other aspects of nutrition competencies and attitudes (such as responsibility of food decisions, consumption of home-cooked meals and consumption of simpler meals when alone/with spouse) differ significantly between the genders. Education was associated with the self-reported ability to seek and verify nutrition information while caregiving arrangements might have an influence on the responsibility of food decisions and consumption of home-cooked meals among the study population. Moreover, higher nutrition knowledge index is found to be associated with being female, of Chinese ethnicity, self-reported ability to understand nutritional information and having access to help from family/friends.

Males were found to have a lower nutrition knowledge index. They were also more likely to leave food decisions to others, less likely to consume home-cooked meals and more likely to consume simpler meals when alone/with spouse. These suggest that more support might need to be offered to older male adults to improve their nutrition knowledge so that they are able to make appropriate food choices, especially when they are dining out. On the other hand, it remains unclear why males prefer to leave food decisions to others and this could potentially be explored as a future research study.

Education is known to contribute to an individual’s ability to better understand and remember different information [42], and educated adults were suggested to be more motivated and interested in nutritional health [19, 29]. This is in line with our bivariate analyses which showed that older adults with higher education levels had a higher proportion with the self-reported ability to seek and verify nutrition-related information compared to those with primary education levels. The current study did not find an association between age and nutrition knowledge index. Previous studies on older adults noted that nutrition knowledge decreased as age increased [19, 20, 43]. However, Parmenter et al. found a nonlinear trend between different age cohorts and nutrition knowledge scores and suggested the influence of upbringing and lifestyle on nutrition knowledge [44]. Lin and Lee reported that while dietary habits of the older Taiwanese were strongly influenced by Chinese traditional food-related beliefs (i.e., heaty foods, cold foods, etc.), education had an effect on these beliefs. Higher educated individuals were less likely to report their food choices being influenced by these traditional beliefs. The cohort in the present study is highly educated, 13.0% had education levels of University and above compared to 5.8% of Singapore’s general population aged 65 years and above. Our findings on the lack of association between age and nutrition knowledge could have been due to the higher education levels, which could have equipped individuals with better abilities for seeking and verifying nutrition information.

Older adults who are cared for by family or others reported a higher likelihood of consuming home-cooked meals compared to those who are self-reliant for care. Moreover, the former group is more likely to leave food decisions to the discretion of others compared to those who are self-reliant for care. These suggests that employed caregivers or family members living together with older adults should be included in nutritional education programs targeted at the older adults as they play an important role in ensuring the nutrition health of their older dependents.

Findings on the factors associated with higher nutrition knowledge index from this study highlighted that older male adults may need more assistance to improve their nutrition knowledge compared to the female counterparts. Our results which showed that older female adults had higher nutrition knowledge than males are similar to what was reported by Aihara and Minai in the Asian older adults population and by Shatenstein et al. in the Western older adults population [30, 31]. This could be due to females taking on more domestic responsibilities, including meal preparation and food choices [45], and is thus more likely to be familiar with nutrition-related information. It is also important to note the self-reported ability to understand nutrition information and having access to family and friends for help are contributing factors to the learning and encoding of nutrition knowledge, therefore yielding a higher nutrition knowledge index. Nutritional educational programs which offer small incremental blocks of information content were suggested to booster learning and maximize retention of nutrition among adults [46]. Such an approach may be useful in designing of the nutrition strategies to mitigate age-related declines in comprehension among older learners. The role that social networks play in the nutritional well-being of older adults has been well established in literature [47–49]. In this study, about 14% to 20% of older adults reported obtaining their nutrition information through their social networks. This implies that interventions for improving nutrition literacy should incorporate group-based activities that allows for social interactions and community bonding.. Though majority of the participants in this study reported that nutritional value as a crucial factor influencing their food choices, almost half of the study cohort were more likely to eat simpler meals (i.e., biscuits and milo/pickled vegetables with rice) when dining alone or with spouse. This again highlights the importance of social networks in influencing the nutrition behavior of older adults where they eat more and eat better in social environments with family or friends [50]. Community-based programs that enable older adults to gather in common social spaces to partake in meals together which concurrently allow for the formation of an informal social network may be effective targeted approach to help improve nutritional intake, particularly among older adults who tend to eat alone as a result of poor social network.

Our study found that ethnicity was associated with NKI with older Chinese adults scoring better NKI than others. This finding is similar to that of a previous study conducted in Malaysia, the study’s sample consisted of Malays (46.0%), Chinese (32.0%), Indians (6.0%) and other minority groups (16.0%) aged 60 to 96 years old [28]. The authors found that those of Chinese ethnicity had the highest proportion with good nutrition knowledge compared to the other groups. This suggests that there is a place for targeted and culturally appropriate outreach in order to improve the nutritional knowledge of other ethnic groups.

### Sources of nutrition information

Understanding the sources for nutrition information among the older population is crucial in developing nutrition educational outreach methods. Our study participants reported television (40.0%) as one of the most common sources of information, followed by printed materials like newspapers (39.3%). Similarly, studies on Asian older adults have also found television to be one of the most commonly cited resource of nutritional information, which range from 49.7 to 79.8%, followed by printed materials, social networks and health professionals [20, 30]. Our reported proportion of internet as a source of information as being higher compared to recent studies done in Midwest US on older adults aged 60 years and above (0.4%) [51] and Australia older adults aged 65 years and above (19.0%) [52] was a novel finding. This could be due to the higher education levels and socioeconomic status seen in our participants compared to Singapore’s general population aged 65 years and above. This finding opens up the possibility of internet-based nutrition educational and lifestyle programs, which has shown great potential in increasing access to nutrition information and in improving older adults nutrition knowledge [53, 54]. Additionally these programs are capable of reaching a wide population of individuals at low costs [55].

### Limitations

There are four limitations in this study. Firstly, the participants in this study were relatively healthy and educated with normal nutritional status (MUST = 0). Future studies could examine other older population group such as those at risk of undernutrition. Nevertheless, this is the first study to have looked at this healthy population in Singapore. Secondly, the cross-sectional design means that causal relationship between nutrition knowledge, competencies, attitudes and factors associated with them could not be established. Future studies are needed to understand and confirm the associations in this study. Thirdly, the psychometrics properties of the NLQ have yet to be established, and future studies are planned to validate the questionnaire. Lastly, associations between nutrition knowledge, competencies and attitudes and dietary habits could not be determined due to a lack of dietary intake information. Future studies could capture dietary intake to examine the associations.

## Conclusions

This is the first study to examine the nutrition knowledge, competencies and attitudes among relatively healthy older adults in a multi-ethnic Asian setting. Higher nutrition knowledge levels were found for females, and individuals who were of Chinese ethnicity, a self-reported ability to understand nutritional information, and those who were able to access help from family/friends. Older adults who were cared for by family or others were more likely to consume home-cooked food and were more reliant on others for food decisions. Moreover, this study highlighted the importance of social networks in influencing nutrition knowledge and eating habits. Therefore, our findings provide insights into the design and implementation of nutrition educational programs and intervention programs. Community and clinical nutrition educational programs should be group-based, targeting older adult males, caregivers, as well as those from the minority ethnic groups in Singapore. Interventions targeting healthier eating habits, with the eventual aim of improving the health status of older adults could incorporate community gatherings, and the provisions of nutritious meals.. Future outreach activities could tap on the television and internet for the larger community-dwelling older adult population. Overall, the findings of this study offer relevant insights to policy-makers and health professionals on how to tailor interventions and public health outreach initiatives to specific groups of older people to help optimize their nutritional knowledge among community-dwelling older adults in a multi-ethnic Asian setting. Future research into the community-dwelling older adult population is needed, in terms of examining the associations between nutrition literacy, nutritional status and health outcomes.

## Data Availability

The datasets from this current study are available from the corresponding author on reasonable request.

## Abbreviations

BMI: Body mass index
CCI: Charlson Comorbidity Index
MBI: Modified Barthel index
MUST: Malnutrition UNIVERSAL SCREENING TOOL
NKI: Nutrition knowledge index
NLQ: Nutrition Literacy Questionnaire
